# Design and Validation of a Cyber–Physical Medication Dispensing Platform Integrating Edge AI Verification, Distributed Control, and Cloud Synchronization

**DOI:** 10.3390/s26123823

**Published:** 2026-06-16

**Authors:** Buddharaksa Phatcharasaksakol, Supaphan Sittithanon, Veerinrada Pianapitham, Vipas Chantrapanichkul, Jing Tang, Ratchatin Chancharoen

**Affiliations:** 1 International School of Engineering, Faculty of Engineering, Chulalongkorn University, Bangkok 10330, Thailand; 6538135121@student.chula.ac.th (B.P.); 6538216321@student.chula.ac.th (S.S.); 6538195821@student.chula.ac.th (V.P.); 6538192921@student.chula.ac.th (V.C.); 2 Chulalongkorn School of Integrated Innovation, Chulalongkorn University, Bangkok 10330, Thailand; jing.t@chula.ac.th; 3 Center of Excellence in Disaster and Risk Management Information Systems, Chulalongkorn University, Bangkok 10330, Thailand; 4 Department of Mechanical Engineering, Faculty of Engineering, Chulalongkorn University, Bangkok 10330, Thailand; 5 Center of Excellence for Human-Robot Collaboration and Systems Integration, Chulalongkorn University, Bangkok 10330, Thailand

**Keywords:** cyber–physical systems (CPSs), automated medication dispensing, edge computing, Internet of Things (IoT), smart healthcare systems

## Abstract

Medication dispensing errors remain a significant concern in healthcare systems, particularly in elderly care and long-term medication management, where incorrect medication delivery may compromise patient safety and treatment outcomes. This study presents the design and experimental validation of a cyber–physical medication dispensing platform integrating robotic manipulation, edge AI-based visual verification, distributed motion control, and cloud synchronization. The platform combines a rotary medication storage mechanism, vacuum-based pill handling, a Klipper-based control framework, and a YOLOv8 perception subsystem deployed on a Hailo AI accelerator for real-time edge inference. Experimental evaluation was conducted under controlled laboratory conditions. Using an environment-specific validation dataset, the perception subsystem achieved a precision of 0.627, recall of 0.739, and mAP@0.5 of 0.786. An adaptive verification strategy was subsequently evaluated to improve dispensing verification under varying pill occupancy conditions. End-to-end system testing comprising 80 dispensing trials achieved an overall dispensing success rate of 86.25%, with no incorrect dispensing events observed. The results demonstrate the feasibility of integrating edge AI verification, distributed control, and cloud connectivity within a cyber–physical medication dispensing platform. The presented system provides a foundation for future research on perception-assisted medication dispensing, long-term deployment, and clinical validation in smart healthcare environments.

## 1. Introduction

**Figure 1:**
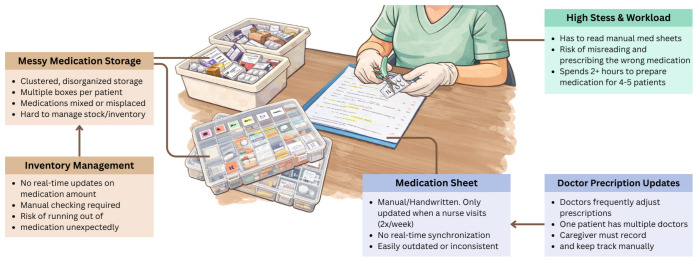
Medication management workflow and associated challenges in elderly care environments, highlighting fragmented processes, manual handling, and the absence of integrated verification and real–time data synchronization.

Medication management in elderly care environments remains a persistent and high-risk challenge, driven by the increasing prevalence of chronic conditions, polypharmacy, and frequent prescription updates [1]. As care complexity grows, the process of preparing, organizing, and administering medication becomes progressively more demanding, often relying on manual workflows that are inherently prone to error [2]. These workflows typically involve multiple stages—prescription transcription, pill sorting, verification, storage, and administration—each of which introduces opportunities for inconsistency, misinterpretation, and delay. In institutional settings where a single caregiver may be responsible for multiple patients, these risks are further amplified by workload pressure and fragmented communication. As illustrated in Figure 1, these fragmented manual processes represent a key source of systemic risk in elderly care medication management.

A substantial body of research has established that medication errors are systemic rather than isolated events, arising from the interaction between human limitations and fragmented workflows [3,4,5]. In response, a range of automated medication management systems has been proposed. Early solutions primarily focus on reminder-based mechanisms and basic dispensing devices that support adherence but provide limited feedback on execution correctness [6,7]. More recent approaches extend these systems through IoT integration, enabling remote monitoring and caregiver notification; however, they typically rely on schedule-driven logic and lack adaptability to real-world variations in pill handling and user interaction [8,9,10]. A recent systematic review further highlights that existing systems often fail to support multi-user environments and lack robust mechanisms for verifying whether dispensing actions have been physically executed as intended [11].

Parallel to these developments, advances in cyber–physical systems (CPSs) and edge computing have enabled tighter integration between sensing, computation, and actuation in healthcare applications [12,13]. In particular, the incorporation of perception into control loops provides a pathway toward verifiable automation, where physical actions can be observed and validated in real time. Deep learning-based computer vision techniques, such as object detection models for pill recognition, have demonstrated promising results under controlled conditions [14]. However, their deployment is often limited to offline analysis or isolated subsystems, with limited integration into real-time operational pipelines.

At the same time, the availability of open-hardware motion control platforms has enabled the development of low-cost and highly programmable automation systems. Prior work has demonstrated that such platforms, originally designed for additive manufacturing, can be repurposed for precision manipulation tasks through appropriate control abstractions [15,16]. Despite these advances, existing solutions typically address mechanical design, perception, or system coordination in isolation. There remains a lack of unified architectures that integrate these components into a coherent framework capable of linking digital prescription data with physically verifiable dispensing actions.

In response to these challenges, this paper presents the design and experimental validation of a cyber–physical medication dispensing platform that integrates robotic manipulation, edge-based visual verification, distributed motion control, and cloud synchronization within a unified architecture. The proposed system combines a Rotary Indexing Storage Assembly and Linear Positioning Stage for structured pill handling, a vacuum-based end effector for medication transfer, and a vision-based verification subsystem implemented using a YOLOv8 model accelerated on a Hailo AI accelerator. Motion control is implemented through a Klipper-based architecture [17], enabling coordinated and deterministic execution of dispensing operations, while a cloud-based synchronization layer maintains consistency among user interfaces, dispensing records, and system states.

The contribution of this work lies in the integration and experimental evaluation of perception, control, communication, and actuation technologies within a single cyber–physical medication dispensing platform. In particular, the vision subsystem is employed as a verification layer to monitor dispensing outcomes and provide operational feedback regarding dispensing success and failure events. Through the combination of edge AI verification, distributed control, and cloud connectivity, the platform provides a testbed for investigating perception-assisted medication dispensing and cyber–physical healthcare automation.

The contributions of this paper are threefold. First, it introduces a unified CPS architecture that bridges digital prescription data and physical dispensing through structured coordination across hardware and software layers. Second, it demonstrates the integration of edge-based vision verification within a deterministic motion control framework, enabling perception-informed validation of dispensing actions. Third, it provides a comprehensive experimental evaluation of system performance, highlighting both the strengths of the proposed approach and the limitations of current perception methods under challenging conditions.

## 2. System Architecture

### 2.1. System Overview

The proposed system is formulated as a cyber–physical system (CPS), defined as an integration of computation and physical processes in which embedded computers monitor and control physical operations through feedback loops [18], consisting of four interconnected layers: hardware, edge computing, cloud coordination, and user interaction. This layered architecture enables the separation of functional responsibilities while maintaining coordinated system-level operation. Such an approach is widely adopted in smart healthcare systems, where sensing, computation, communication, and actuation must operate in a synchronized manner [19].

The hardware layer performs physical actuation and sensing through a modular robotic architecture, enabling deterministic manipulation and structured medication handling [20]. The edge layer provides real-time computation, including perception and control, thereby supporting low-latency decision-making. The cloud layer maintains synchronization of system states and supports IoT-based coordination across distributed clients [21]. The user interaction layer enables caregiver engagement through web-based and mobile interfaces, allowing remote monitoring and control.

**Figure 2:**
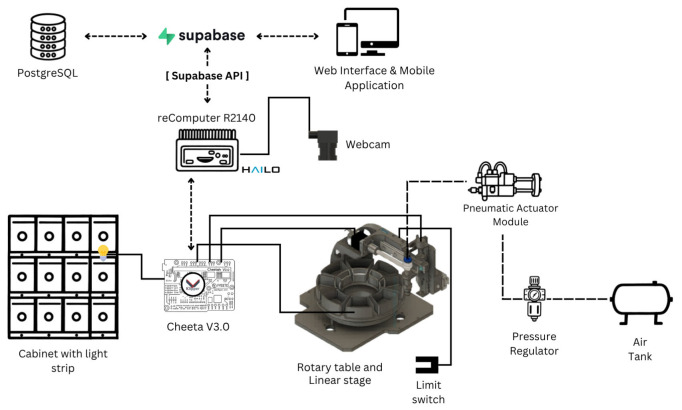
Cyber–physical architecture for medication management and dispensing, illustrating the integration of sensing, control, communication, and actuation to enable coordinated and reliable system operation. Dashed arrows indicate data communication, control commands, and feedback exchange between system components, while dashed lines in the pneumatic subsystem represent compressed-air connections between pneumatic devices. Solid lines represent direct hardware integration and actuation pathways.

A key design consideration is the decoupling of high-level decision-making from time-critical execution. It has been observed that such separation is essential for ensuring reliable operation in safety-critical systems. In the proposed implementation, high-level coordination is performed at the edge and cloud layers, while deterministic actuation is executed locally within the hardware layer. This design enables distributed control systems to maintain real-time performance while supporting scalability and integration with IoT-based healthcare services. The overall architecture is illustrated in Figure 2.

### 2.2. Hardware Layer

The hardware layer constitutes the physical embodiment of the proposed system and is responsible for executing dispensing operations and acquiring sensory data. It is organized as a modular robotic architecture, comprising three main functional groups: mechanical actuation, pneumatic manipulation, and sensing. This structured design enables reliable and repeatable operation while supporting system-level integration within the cyber–physical framework.

The mechanical subsystem includes a Rotary Indexing Storage Assembly and a Linear Positioning Stage, which together form a structured two-degree-of-freedom addressing mechanism. The rotary mechanism provides indexed access to multiple medication compartments, while the linear stage enables precise positioning of the end effector during pick-and-place operations. Actuation is achieved using stepper motors controlled through a 3D printer control board and associated motor drivers [22], allowing repeatable motion along predefined trajectories.

Pill manipulation is implemented using a vacuum-based end effector, consisting of a Vacuum Gripper, Vacuum Pad, and Vacuum Gripper Driver, supported by a Pneumatic Actuator Module and Air Tank for stable pressure regulation. This approach enables non-invasive handling of pills with varying geometries [23], which is particularly important in healthcare applications where maintaining physical integrity is essential.

The sensing subsystem includes limit switches for positional referencing and safety, as well as a camera for visual perception. The camera provides input to the vision-based verification pipeline, enabling detection, localization, and counting of pills during operation [24]. It has been shown that integrating perception at this stage allows the system to validate physical outcomes, thereby enhancing overall reliability.

The computational hardware follows a distributed configuration [20], supporting the implementation of distributed control systems. The Raspberry Pi 5 functions as the edge computing platform, responsible for high-level coordination, task planning, and execution of perception algorithms. Time-critical control tasks, including stepper motor pulse generation and actuator coordination, are executed by the STM32F446RCT6 under the Klipper framework. In addition, a Hailo AI Accelerator is integrated [25], enabling efficient real-time inference.

Collectively, this heterogeneous hardware configuration enables a clear separation between high-level computation and deterministic actuation. Such an arrangement supports system-level integration of perception, control, and actuation [26], and provides a scalable foundation for cyber–physical systems and intelligent control systems in smart healthcare applications.

### 2.3. Edge Layer

The edge layer serves as the computational core of the system, enabling real-time processing and coordination between perception and control. It is implemented on the Raspberry Pi 5 and operates as the supervisory node within the distributed control architecture. It is observed that the edge layer performs multiple functions, including task planning, command generation, and execution of the vision-based inference pipeline [27]. Object detection is implemented using a YOLOv8 model, enabling low-latency perception suitable for real-time verification.

Control coordination is achieved through the Klipper-based framework, which separates high-level computation from real-time execution. The edge node executes trajectory planning and system coordination, while the microcontroller performs deterministic actuation. This architecture may be interpreted as an intelligent control system, in which perception, planning, and execution are integrated within a hierarchical structure. The edge layer also interfaces with the cloud through IoT communication protocols, enabling synchronization of system states and remote interaction.

### 2.4. Cloud Layer

The cloud layer functions as the coordination and data management component of the system, maintaining a consistent representation of system states across distributed interfaces and supporting remote operation. All system data, including medication records, dispensing schedules, and operational logs, are stored in a centralized database to reduce the risk of data divergence across distributed components [28]. Real-time synchronization is achieved through an event-driven architecture that propagates state changes to all connected devices. The cloud layer additionally supports auxiliary services such as notification delivery and AI-assisted functionalities, but does not perform time-critical control tasks; deterministic execution remains with the edge and hardware layers.

### 2.5. Data and Control Flow

The system operates through an event-driven data and control flow that links user interaction, cloud coordination, edge computation, and hardware execution. It can be observed that this structure enables a closed-loop CPS, where perception is used to validate physical outcomes.

A typical operation begins with a user request initiated through the web or mobile interface. This request is transmitted to the cloud layer and subsequently propagated to the edge system. The edge layer translates the request into a sequence of control commands, which are executed by the hardware layer through the distributed control framework.

During execution, the camera captures visual data, and the edge AI module performs detection and verification. The outcome of the operation is evaluated and transmitted back to the cloud layer, where it is recorded and synchronized across user interfaces.

This bidirectional flow ensures that each dispensing action is both executed and verified, thereby enhancing system reliability, and represents a key characteristic of cyber-physical systems in smart healthcare, where physical actions must be continuously validated against digital intent.

## 3. Mechanical System Design

This section describes the mechanical design of the proposed medication dispensing system. The system has been developed to enable structured, repeatable, and reliable handling of individual pills within a compact and integrated platform. In line with the overall cyber–physical system (CPS) architecture, the mechanical design focuses on deterministic addressing, controlled manipulation, and consistent transfer of medication units from storage to the output location.

It has been widely reported that mechanical uncertainty can significantly affect the reliability of automated dispensing systems. Therefore, the present design prioritizes alignment consistency, repeatability, and structural simplicity. This approach is considered particularly suitable for smart healthcare applications, where correctness and traceability are more critical than high throughput.

### 3.1. Design Overview

The mechanical system is centered on a modular robotic architecture that integrates rotational indexing, linear positioning, and vacuum-based manipulation.

**Figure 3:**
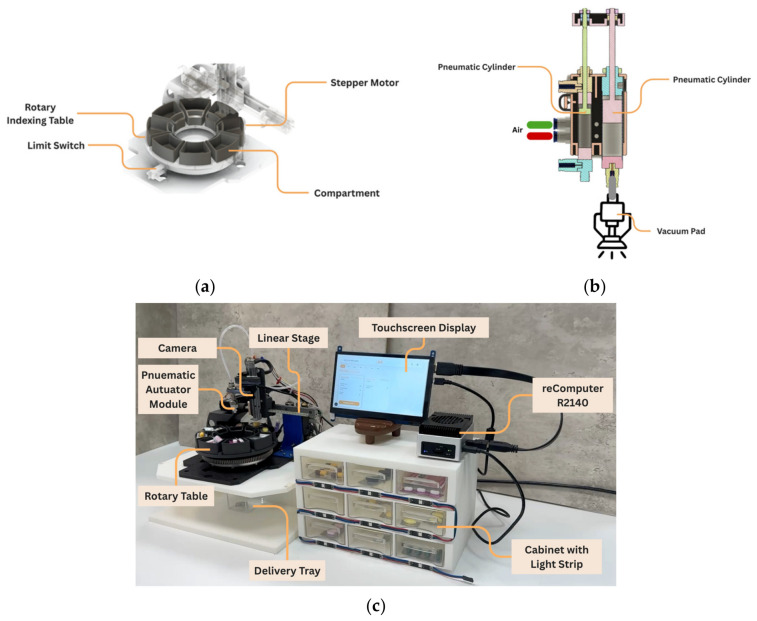
Mechanical architecture of the dispensing system integrating rotary storage, linear positioning, and vacuum-based manipulation. (**a**) Rotary indexing mechanism with radially arranged compartments, where each slot is assigned a predefined angular position for deterministic medication addressing. (**b**) Vacuum-based pick-and-place sequence illustrating pill acquisition, transfer, and controlled release. (**c**) Photograph of the developed hardware prototype, including the automated rotary indexing table and the storage cabinet with integrated light guidance.

The mechanical system integrates three main subsystems, as illustrated in Figure 3: a Rotary Indexing Storage Assembly, a Linear Positioning Stage, and a vacuum-based end effector [29]. This assembly is coupled with a Linear Positioning Stage, forming a two-degree-of-freedom (2-DoF) addressing mechanism that allows each compartment to be accessed through a predefined combination of rotational and translational coordinates [30]. Such a configuration eliminates the need for dynamic search or continuous sensing during operation.

Motion within the system is driven by stepper motors controlled via a 3D printer control board and associated motor drivers, enabling precise and repeatable positioning. The mechanical structure is designed to maintain alignment between the storage compartments, gripper, and delivery pathway, ensuring that each dispensing cycle follows a consistent trajectory. The delivery tray serves as the final interface for pill output, completing the transfer process.

### 3.2. Rotary Storage and Indexing

The rotary storage mechanism is the core of the medication handling system, providing structured organization and deterministic access to compartments. The rotary indexing table comprises radially arranged compartments, each assigned a fixed angular position, enabling direct mapping between digital medication schedules and physical storage locations without the need for dynamic search or sensing. A stepper motor drives the rotation, ensuring precise and repeatable angular positioning.

The table completes a full 360-degree rotation, with eight compartments spaced at 45-degree intervals. Instead of returning to a home position between cycles, the system uses relative positioning, where angular displacement is continuously accumulated. This allows rotation beyond 360 degrees, eliminating unnecessary return motion and reducing cycle time. The target compartment is reached via the shortest angular path from the current position, improving efficiency in sequential multi-medication dispensing by minimizing motion overhead and mechanical wear.

The circular configuration enhances spatial efficiency and maintains balanced mass distribution, while the fixed compartment design reduces the risk of mixing or misplacement.

### 3.3. Linear Positioning Stage

The Linear Positioning Stage provides controlled translational motion to complement the rotary indexing mechanism, forming a two-degree-of-freedom addressing system. While the rotary table aligns the selected compartment in the angular direction, the linear stage enables precise positioning of the end effector along a predefined axis.

The stage is actuated by a stepper motor, allowing fine control over displacement and ensuring repeatable positioning across multiple cycles. It has been observed that decoupling rotational and translational motion simplifies kinematic control while maintaining sufficient flexibility to access all compartments.

This configuration enables accurate alignment between the vacuum end effector and the target compartment, which is critical for successful pick-and-place operations. The use of predefined coordinate mappings further enhances system robustness, as each compartment can be addressed using fixed positional parameters without the need for real-time adjustment.

### 3.4. Vacuum-Based Gripper

Pill manipulation is achieved through a vacuum-based end effector, comprising a Vacuum Gripper and Vacuum Pad actuated by a Pneumatic Actuator Module. This approach enables non-invasive handling of pills with varying sizes and shapes while minimizing mechanical contact and reducing the risk of damage. The integration of pneumatic actuation further provides stable and controllable suction forces, which are essential for consistent pick-and-place operations.

This pick-and-place mechanism provides precise control over the transfer process and reduces the likelihood of unintended multiple pickups. The vacuum-based approach offers several advantages, including reduced mechanical complexity and minimized contact forces, which help prevent damage to the pills.

However, it has been noted that the effectiveness of the gripper is influenced by factors such as surface area, pill orientation, and sealing conditions. These factors may introduce variability in pickup reliability. In the current prototype, a fixed vacuum source is utilized, and the suction force is not actively regulated according to pill size, shape, or surface characteristics.

### 3.5. Camera-to-Workspace Calibration

To establish correspondence between the perception subsystem and the dispensing mechanism, a camera-to-workspace calibration procedure was performed. The proposed platform employs a fixed overhead camera positioned above a structured Cartesian dispensing workspace; therefore, a conventional hand–eye calibration approach is not required. Instead, calibration was achieved through image-to-workspace coordinate transformation using predefined reference points located within the dispensing tray. The pixel coordinates extracted from the camera image were mapped to physical workspace coordinates through a planar geometric transformation, enabling detected pill locations to be translated into actionable target positions for the manipulation subsystem.

This calibration procedure establishes a common reference frame between the vision and actuation modules, allowing the robotic system to accurately associate detected pills with corresponding dispensing locations. The resulting coordinate mapping is subsequently used by the motion planning algorithm to generate positioning commands for the linear stage and vacuum-based end effector. The effectiveness of the calibration framework was validated through the localization experiment presented in Section 7, which yielded a mean localization error of 8.3 pixels. This result indicates that the perception subsystem provides sufficiently accurate spatial information for reliable pill localization, pickup, and dispensing operations within the proposed cyber–physical medication dispensing system.

### 3.6. Structural Integration

The mechanical components are integrated within a unified structural framework that ensures stability, alignment, and accessibility. Maintaining consistent spatial relationships between the rotary indexing mechanism, linear positioning stage, and vacuum-based gripper is critical for repeatable operation. The layout separates moving mechanical components from electronic modules to reduce interference and simplify maintenance, while structural rigidity is prioritized to minimize deflection and vibration. Access points are incorporated for loading, unloading, and maintenance, and the delivery pathway provides a clear and controlled output for dispensed pills.

### 3.7. Design Considerations

Several design considerations have been taken into account to ensure the practicality and reliability of the mechanical system. First, the design prioritizes repeatability over speed, recognizing that consistent and accurate dispensing is more critical than high throughput in healthcare applications. Second, mechanical complexity is minimized by reducing the number of moving parts and simplifying motion coordination, thereby improving robustness and ease of maintenance.

Modularity is another key consideration. It can be seen that individual components, such as the rotary compartments and gripper assembly, can be accessed and replaced independently. This facilitates maintenance and future upgrades without requiring complete system disassembly.

Despite these advantages, certain limitations remain. The performance of the vacuum-based end effector may vary depending on pill geometry and surface conditions, potentially affecting pickup reliability. In addition, the system relies on predefined compartment positions and does not dynamically adjust to variations in pill placement within compartments.

These limitations highlight the importance of integrating perception-based verification within the system. By combining deterministic mechanical design with edge-based AI perception, the proposed architecture aims to enhance overall system reliability within a broader IoT-enabled cyber–physical system for smart healthcare applications.

## 4. Control Framework

This section presents the control framework of the proposed system, which coordinates user interaction, edge computation, and real-time actuation within a unified cyber–physical system (CPS). In contrast to Section 2, which describes the architectural organization, this section focuses on the execution of control tasks, including motion planning, command generation, and system coordination. The proposed framework adopts a distributed control system structure that separates high-level computation from time-critical execution, while maintaining synchronized interaction across all system layers.

### 4.1. Control Architecture Overview

The control framework coordinates high-level task execution with deterministic low-level actuation within the CPS architecture [31] through a hierarchical organization that separates decision-making, communication, and real-time motion control. The structure consists of three levels—user interaction, edge coordination, and real-time execution—allowing complex task planning to be decoupled from time-critical execution, which is essential in safety-critical healthcare applications [32].

At the user interaction level, dispensing requests are initiated through the Web Interface, Mobile Application, or Touchscreen Display. These requests are defined based on medication schedules and patient-specific requirements. The system adopts an event-driven approach, whereby each user command triggers a sequence of coordinated actions across computational and physical subsystems. Requests are transmitted to the edge layer through IoT-based communication mechanisms, ensuring seamless integration between distributed interfaces.

**Figure 4:**
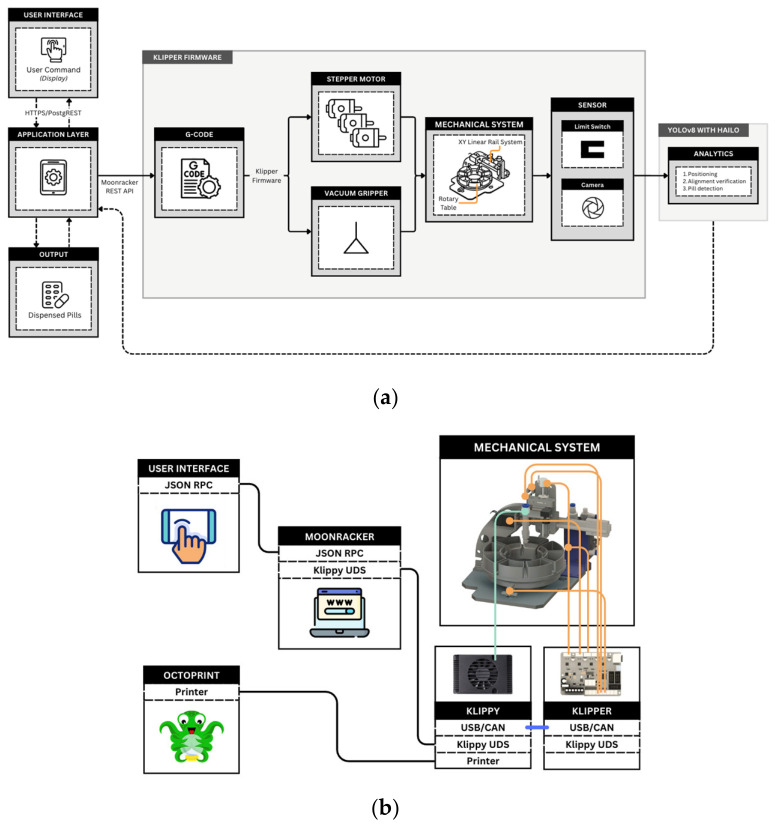
Control architecture of the automated medication dispensing system and its hierarchical execution framework. (**a**) System-level control pipeline illustrating the flow from user interaction to physical actuation and feedback. User commands are issued via a web interface and translated into G-code through the Moonraker API. Klipper firmware executes deterministic motion control of the rotary table, linear stage, and vacuum gripper, while sensor inputs and camera feedback enable system monitoring and vision-based verification using a YOLOv8 module, forming a closed-loop cyber–physical system. (**b**) Hierarchical control framework based on Klipper firmware, where high-level task planning and motion generation are executed on an edge computing host, and time-critical actuation is handled by the microcontroller. This host–MCU separation ensures deterministic execution and reliable coordination of dispensing operations. Black lines represent software communication pathways between system components, the blue line denotes USB/CAN communication between the Klippy Host and Klipper MCU, orange lines indicate MCU-level control and feedback connections to the dispensing hardware, and the green line represents a host-side hardware interface connected directly to the mechanical subsystem.

At the edge coordination level, the system performs task planning, command generation, and system state management. This layer is implemented on the edge computing platform (reComputer R2140), where the Klipper (v0.13.0) host software functions as the supervisory controller. Dispensing requests are translated into structured task sequences, decomposed into motion primitives—rotary indexing, linear positioning, and end-effector actuation—encoded as G-code and communicated via the Moonraker API. The host–MCU separation is illustrated in Figure 4b.

At the real-time execution level, time-critical operations are carried out by the microcontroller under the Klipper firmware framework, including stepper motor pulse generation, actuator coordination, and synchronization of multi-axis motion. The firmware ensures deterministic execution of predefined trajectories, supported by homing procedures using limit switches for positional referencing and safety.

The hardware layer performs physical actuation through coordinated control of the stepper motors and the pneumatic actuator module, enabling precise motion of the Rotary Indexing Table, Linear Positioning Stage, and Vacuum Gripper. In parallel, the perception subsystem operates as a verification layer within the control loop [33], with detection results used to validate dispensing outcomes via a YOLOv8-based model on the Hailo AI accelerator, as shown in Figure 4a. The full perception integration is detailed in Section 4.5.

### 4.2. Klipper-Based Distributed Control

The motion control subsystem is implemented using a Klipper-based architecture [16], which represents a transition from monolithic embedded control toward a distributed cyber–physical control paradigm. Conventional firmware typically executes motion planning and actuation within a single microcontroller, thereby limiting scalability and integration with higher-level algorithms.

In contrast, the Klipper framework adopts a heterogeneous computing model in which a host edge device is coupled with a dedicated microcontroller. The edge device performs computationally intensive tasks, including G-code parsing, trajectory planning, and system coordination, while the microcontroller executes time-critical operations with deterministic timing.

It can be observed that this separation introduces a clear distinction between non-real-time and real-time processes. From a systems perspective, the architecture may be formalized as a distributed control system, where the edge node functions as a supervisory controller and the microcontroller operates as a deterministic execution unit.

Such an arrangement enables system-level integration of perception, control, and actuation, and provides a scalable foundation for modular robotic architectures. Furthermore, the use of middleware interfaces, such as the Moonraker API, supports both user-level interaction and programmatic control, thereby enabling integration with IoT services and higher-level decision-making processes.

### 4.3. Task Planning and G-Code Generation

Task planning is performed at the edge layer, where dispensing requests are translated into structured sequences of motion primitives including rotary indexing, linear positioning, vertical pick-and-place motion, and pneumatic activation. These are encoded as G-code instructions defining positional targets, motion parameters, and execution order. Using G-code as an intermediate representation ensures compatibility with established motion-control frameworks and simplifies integration with the Klipper-based execution layer, allowing complex dispensing operations to be expressed in a structured and modular manner.

### 4.4. Actuation Sequence

The dispensing process is implemented as a deterministic actuation sequence in which each cycle follows a predefined pipeline, ensuring consistency across repeated operations.

First, the rotary indexing mechanism aligns the target compartment with the pickup position. Next, the linear positioning stage moves the end effector into alignment with the selected pill. The vacuum gripper then descends, and suction is applied to lift the pill from the compartment. The gripper subsequently transports the pill to the delivery location, where it is released into the output tray.

This sequence is encapsulated as a macro within the control framework, enabling it to be executed as a single atomic operation. It may be argued that such encapsulation reduces the likelihood of intermediate failures and improves overall system reliability.

### 4.5. Perception-Integrated Control

The integration of visual perception into control loops has been explored in robotic manipulation tasks, where vision is used to guide grasping and interaction with physical objects. In addition to deterministic motion control, the proposed system incorporates a perception-based verification layer within the control loop. The camera captures visual data during operation, and the edge AI module performs detection and localization using a YOLOv8-based model.

It can be observed that perception is not used as a direct control signal for continuous motion adjustment. Instead, it functions as a verification mechanism that evaluates the outcome of each dispensing operation. This approach allows the system to maintain deterministic execution while introducing an additional layer of reliability.

The perception outputs, including detection confidence and object count, are used to validate whether the intended action has been successfully completed. In cases where inconsistencies are detected, the system may trigger re-acquisition or user notification.

This design reflects a verification-driven control strategy, which is particularly suitable for safety-critical applications such as medication dispensing. It has been suggested that such approaches provide a balance between robustness and system complexity.

### 4.6. Web-Based Interface and IoT Interaction Layer

The system incorporates a browser-based interface to enable user interaction, monitoring, and control within the proposed IoT-enabled cyber–physical system (CPS). The Web Interface communicates with the motion control subsystem through the Moonraker API, which provides a RESTful interface for dispatching commands and retrieving system state information.

It can be observed that this architecture decouples the user interaction layer from the underlying hardware and control subsystems, allowing control logic to be implemented independently of the physical actuation layer [34]. Such separation is particularly important in distributed systems, where user interaction, computation, and execution may occur across different nodes with varying temporal constraints.

**Figure 5:**
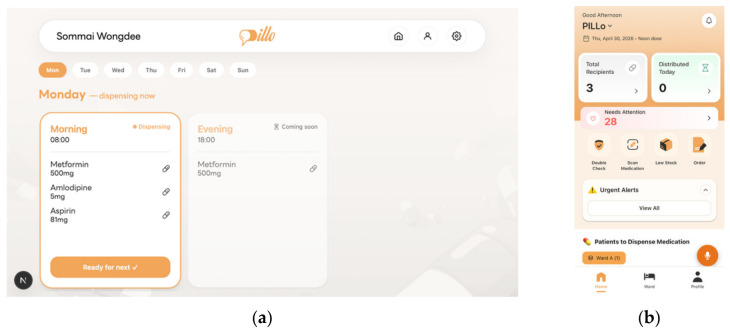
PILLo user interface across client platforms. (**a**) Web-based kiosk interface (Next.js) showing an active Monday morning dispensing session with real-time execution status and caregiver confirmation control. (**b**) Mobile caregiver application (Expo · React Native) displaying the home dashboard with live dispensing summary, urgent alert feed, and quick-action shortcuts for medication scanning and stock management.

Through the interface, users are able to initiate dispensing operations, monitor execution progress, and access system feedback in real time. The web interface is implemented using Next.js [35] (TypeScript [36], Tailwind CSS [37]), deployed on the edge computing node (reComputer R2140) and accessed via Chromium in kiosk mode for local touchscreen operation. The mobile application is developed using Expo [38] (React Native 0.81.5), enabling caregiver interaction from remote devices. Both interfaces are shown in Figure 5. Both clients synchronize system state through Supabase [39] (PostgreSQL with Realtime subscriptions), which serves as the cloud coordination layer. The use of these web technologies ensures accessibility across heterogeneous devices, thereby supporting seamless integration with IoT-based healthcare platforms.

In addition to command issuance, the interface also serves as a visualization layer for system state, including execution status and perception-based verification outcomes. This establishes a bidirectional interaction channel in which user input influences physical execution, and execution results are propagated back to the user, forming a closed-loop interaction within the CPS.

Complementary interaction is supported through a Mobile Application and Touchscreen Display, enabling both remote and local control within a unified framework. It may therefore be argued that the interface layer functions not merely as a user interface, but as an interaction node within a distributed control system, facilitating coordination between human operators, edge computation, and physical actuation.

### 4.7. Safety and Reliability Mechanisms

Several mechanisms are incorporated to ensure safe and reliable operation. At the hardware level, limit switches are used to define reference positions and prevent motion beyond physical boundaries. These constraints are enforced within the control configuration to ensure safe operation.

At the software level, structured macros are used to ensure that dispensing sequences are executed atomically. This reduces the likelihood of partial execution due to communication interruptions or timing inconsistencies.

An emergency stop mechanism is also implemented, allowing immediate termination of motion in the event of abnormal conditions. In addition, system-state monitoring is supported through the Moonraker API, enabling real-time feedback on execution status.

Finally, the integration of perception-based verification provides an additional layer of reliability. It can be argued that combining deterministic actuation with verification-driven validation enhances overall system robustness, particularly in IoT-enabled smart healthcare environments.

## 5. Vision-Based Verification

This section presents the vision-based verification module, which provides a perception-driven validation layer within the proposed cyber–physical system (CPS). The module is designed to observe and evaluate the outcome of dispensing operations in real time, thereby complementing deterministic actuation with perception-based feedback.

It has been widely recognized that open-loop automation systems are inherently limited in their ability to guarantee correct physical execution. Therefore, the integration of perception within the control loop is considered essential for improving system-level reliability. In the proposed implementation, the vision module establishes a verification-driven control paradigm, in which physical actions are continuously assessed against expected outcomes.

### 5.1. Vision System Overview

The vision-based verification module is introduced to provide a perception-driven validation mechanism within the CPS, ensuring that dispensing operations are executed as intended. In contrast to purely deterministic actuation, this module enables the system to observe and evaluate the outcome of each operation.

**Figure 6:**
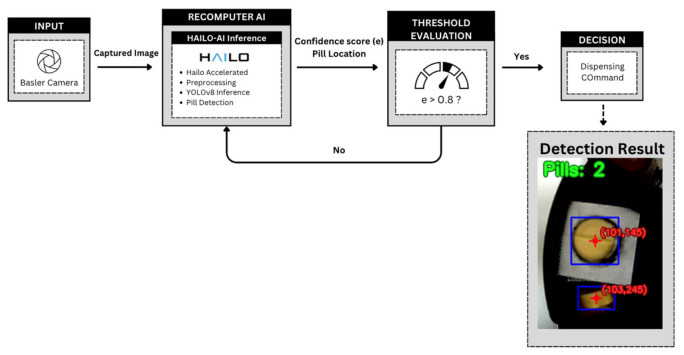
Vision-based verification pipeline for dispensing validation. The pipeline includes image capture, YOLOv8 inference on the Hailo edge accelerator, confidence-based validation, and action triggering. Detected pills are localized and counted to verify dispensing outcomes. The right panel shows a real-time detection example.

The verification process is based on a camera-centered perception pipeline integrated with an edge-based artificial intelligence model, as illustrated in Figure 6. Visual data captured from the dispensing region are processed locally on the edge computing platform using a YOLOv8-based detection model accelerated by dedicated hardware. It can be observed that this enables low-latency inference, allowing verification to be performed in real time without reliance on cloud-based processing.

The primary function of the module is to detect, localize, and count pills within the field of view. These outputs are used to verify key stages of the dispensing process, including pickup, transfer, and release. Detection results are evaluated using a confidence-based criterion, allowing the system to distinguish between valid and uncertain observations.

By integrating this perception layer within the control framework, the system establishes a perception–action loop, in which execution outcomes are monitored and validated. It may be argued that this approach transforms the system from purely deterministic execution to perception-informed operation, thereby enhancing overall reliability.

### 5.2. Image Acquisition and Preprocessing

Image acquisition is performed using a fixed overhead camera positioned above the dispensing region. The camera is mounted at a predefined distance and orientation to ensure a consistent field of view across all operations. It has been shown that maintaining a fixed imaging configuration reduces variability and improves repeatability in perception tasks.

To minimize the effects of environmental variation, illumination conditions are maintained as constant as possible. However, minor variations due to shadows and reflections may still occur and must be accounted for during processing.

Captured images are transmitted directly to the edge computing platform for local processing. Prior to inference, each image undergoes a preprocessing stage, including resizing the required input resolution, normalization of pixel values, and basic filtering where necessary. These steps standardize the input data and improve robustness against variations in lighting and surface properties.

### 5.3. YOLOv8 Detection (Edge AI)

The perception subsystem was implemented using a YOLOv8 object detection model deployed on a Hailo AI accelerator for real-time edge inference. The model was initially trained using the publicly available Pills Detection Dataset developed by Alexander Y. Y. [40]. This dataset combines multiple pill-detection datasets that were re-annotated and converted into YOLO-compatible object detection format, providing a large collection of pill images with bounding-box annotations. The dataset contains more than 12,000 annotated images and over 140,000 pill instances acquired under diverse imaging conditions, including variations in pill orientation, arrangement, and visual appearance.

All annotations were stored in the standard YOLO object detection format, where each object is represented by a class identifier and a normalized bounding box. This format enables efficient training and deployment within the Ultralytics YOLOv8 framework.

To improve model robustness and generalization, the default augmentation pipeline provided by the Ultralytics framework was employed during training. The applied augmentation techniques included image scaling, translation, horizontal flipping, brightness adjustment, color-space variation, and mosaic augmentation. These augmentations increased visual diversity within the training dataset and improved detection performance under varying operating conditions encountered during real-world dispensing tasks.

The YOLOv8 training procedure followed a supervised learning approach using the publicly available dataset as the primary training source. The resulting model was subsequently optimized and deployed on the Hailo AI accelerator to enable low-latency inference within the edge-computing architecture. This deployment strategy allows pill verification to be performed locally without dependence on cloud-based processing, thereby reducing communication latency and improving operational reliability.

To evaluate practical performance within the proposed medication dispensing platform, an additional environment-specific tray-based dataset was collected directly from the dispensing hardware. A controlled pill-counting experiment was conducted using tray configurations containing one to nine pills. For each pill quantity, ten images were captured under consistent environmental conditions, resulting in a total of 90 images. The camera position, lighting conditions, and tray geometry were maintained throughout data collection to ensure experimental consistency.

The environment-specific dataset was used to assess the domain gap between public training data and actual dispensing conditions. In particular, the dataset captures operational characteristics that are not fully represented in public pill datasets, including fixed camera viewpoints, dispensing-tray geometry, partial occlusion, and pill overlap. Consequently, this dataset provides a more realistic evaluation of perception performance within the intended healthcare deployment scenario.

The trained model was subsequently evaluated using a separate validation dataset collected from the dispensing platform. The resulting detection performance and verification accuracy are presented and discussed in Section 7.

### 5.4. Confidence-Based Verification

A confidence-based verification mechanism is employed to filter detection results and ensure that only reliable outputs are used for decision-making. Each detected object is associated with a confidence score e, representing the likelihood that the object corresponds to a pill.

A predefined threshold of e > 0.8 is applied, and only detections exceeding this value are considered valid. If the condition is satisfied, the system proceeds to generate a dispensing command. If the confidence score falls below the threshold, the detection is rejected and the image is re-submitted to the Hailo inference stage for re-acquisition, forming a feedback loop until a valid detection is confirmed or a failure condition is reached. It has been suggested that such conservative filtering reduces the likelihood of false positives, which is particularly important in safety-critical applications such as medication dispensing.

In addition to individual detection validation, the total number of accepted detections within a frame is compared with expected values. This enables the identification of anomalies, such as missing or multiple pills, and provides a simple yet effective mechanism for verifying dispensing correctness.

However, it can be observed that this approach introduces a trade-off between precision and recall. A higher threshold improves reliability but may increase under-detection, particularly in scenarios involving occlusion or overlapping objects.

### 5.5. Integration with Control Framework

The vision-based verification module is integrated into the control framework as a validation layer rather than a direct control mechanism. Detection outputs, including centroid coordinates and verification status, are transmitted to the edge control logic, where they are used to assess the outcome of actuation steps.

In the current implementation, motion execution is primarily governed by predefined trajectories within the Klipper-based control system. The perception module complements this by providing post-action verification, confirming whether the intended operation has been successfully completed.

For spatial alignment tasks, detected centroid coordinates may be mapped from image space to the physical coordinate system through calibration. This mapping enables alignment between perception and actuation subsystems, supporting accurate positioning of the vacuum gripper.

Overall, this integration establishes a perception–action loop in which visual feedback enhances system reliability without significantly increasing control complexity. It may be argued that such an approach provides a practical balance between deterministic execution and adaptive verification.

### 5.6. Limitations and Failure Modes

Despite its effectiveness, the vision-based verification module exhibits several limitations that affect overall system performance. One of the primary challenges is reduced detection accuracy in scenarios involving occlusion or overlapping pills. In densely populated compartments, the model may fail to distinguish individual objects, leading to under-detection. This limitation is consistent with well-documented challenges in object detection under dense and occluded conditions [41].

Lighting conditions and surface reflections also influence detection performance. Variations in illumination may degrade image quality, thereby affecting both detection confidence and localization accuracy. Although preprocessing mitigates some of these effects, complete robustness under all conditions is not achieved.

Another limitation arises from the use of a fixed camera viewpoint. While this simplifies system design and ensures consistency, it restricts the ability to observe objects from multiple perspectives, which may be necessary in more complex environments.

Finally, the use of a static confidence threshold may not be optimal across all scenarios. A threshold that is too high may result in missed detections, while a lower threshold may introduce false positives. This trade-off highlights the need for adaptive or context-aware thresholding strategies in future work.

These limitations suggest that perception performance remains a key constraint in the overall system. Therefore, it may be argued that further improvements, such as enhanced datasets, multi-view sensing, or sensor fusion, are required to achieve robust deployment in real-world smart healthcare environments.

## 6. IoT Synchronization and Data Consistency

The cloud layer of the PILLo system serves as the central coordination and intelligence hub, responsible for maintaining consistent system state across distributed clients, orchestrating hardware actuation, and supporting AI-assisted caregiver interaction. Building on the four-layer architecture described in Section 2, this section details the specific implementation of cloud services, client interfaces, and synchronization mechanisms that enable reliable multi-client operation in a real-world care environment.

**Figure 7:**
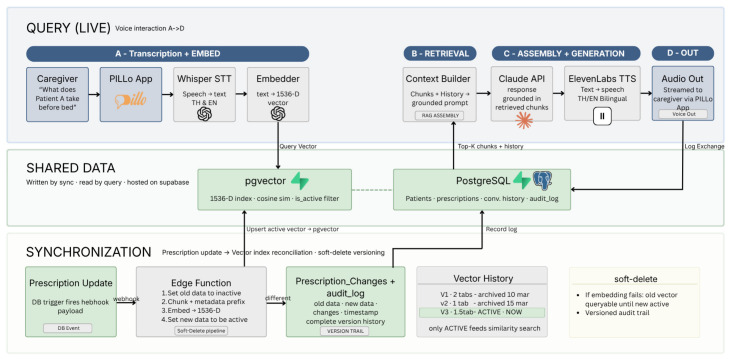
System-level cyber–physical architecture of the PILLo platform. All system states and commands are routed through a centralized Supabase database, ensuring a single source of truth. Downward information flow translates caregiver intent into hardware actuation, while upward feedback propagates real-time system state and verification results across distributed components. Functional layers are distinguished by color: the live voice-query pipeline (blue, stages A–D), the shared Supabase data layer (green, pgvector and PostgreSQL), and the background synchronization pipeline (pale, soft-delete vector reconciliation). Solid arrows denote runtime data flow, with edge labels indicating the payload exchanged at each step (e.g., query vector, top-K retrieved chunks, log records). The dashed connector between pgvector and PostgreSQL indicates co-location within a single Supabase instance rather than a runtime call.

A key architectural decision is the adoption of Supabase as the sole synchronization hub, as illustrated in Figure 7. All components—mobile applications, web interfaces, and hardware controllers—interact exclusively through this shared data layer, with direct device-to-device communication intentionally avoided. This design enforces three critical system properties: consistency, achieved through a single source of truth for all system states [42]; auditability, ensured by persistent transaction logging with temporal metadata [43]; and resilience, maintained through operation queuing that preserves commands issued during network interruptions.

### 6.1. Client Layer

The client layer provides the primary human–system interface through two complementary applications tailored to distinct operational contexts. The mobile caregiver application is designed for distributed use, supporting medication registration, dispensing scheduling, and real-time monitoring from remote devices. It incorporates two AI-assisted capabilities: a drug-label recognition module that performs vision-based parsing of medication packaging to extract structured prescription data, and a voice interaction module built on a retrieval-augmented generation (RAG) pipeline that enables hands-free access to patient medication information during clinical workflows.

The machine-side kiosk interface operates locally on the dispensing hardware and is optimized for execution transparency and guided interaction. It provides real-time visualization of active dispensing sessions and employs LED-guided feedback to assist caregivers in correct medication loading, directly reducing the likelihood of slot assignment errors during system setup. Both clients synchronize system state through Supabase real-time subscriptions, enabling immediate propagation of state changes without polling.

### 6.2. Cloud and Service Layer

**Figure 8:**
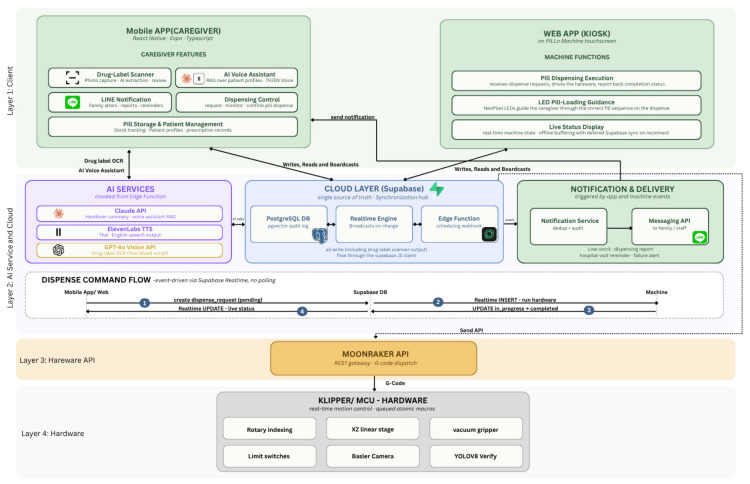
Detailed multi-layer architecture and operational workflow of the PILLo system. (Layer 1—Clients): Mobile caregiver application and kiosk interface providing dispensing control, AI-assisted interaction, and guided loading. (Layer 2—Cloud and Services): Supabase-based synchronization layer with PostgreSQL and real-time broadcasting, integrated with modular AI services for label recognition, voice interaction, and notification handling. (Layer 3—Hardware API): Moonraker REST interface translating cloud-issued commands into G-code for motion execution. (Layer 4—Hardware): Klipper-controlled dispensing mechanism comprising rotary indexing, linear positioning, vacuum gripping, and vision-based verification. Layers are distinguished by color (Layer 1 clients, green; Layer 2 cloud and AI services, blue/purple; Layer 3 hardware API, orange; Layer 4 hardware, grey). Solid arrows denote runtime data flow between components, with edge labels indicating the exchange type (e.g., writes/reads/broadcasts, AI calls, notifications). The numbered steps (1–4) in the Dispense Command Flow trace the event-driven sequence of a single dispense operation, from request creation through hardware execution to live status update.

The cloud layer comprises three functionally distinct components, with the full multi-layer operational workflow illustrated in Figure 8.

The Data and Synchronization Core is built on a PostgreSQL database extended with pgvector for vector search capabilities. This database maintains all persistent system state, including patient records, prescriptions, dispensing logs, and semantic embeddings used for RAG-based retrieval. A real-time broadcasting engine propagates database changes to all subscribed clients, ensuring system-wide consistency across concurrent sessions. Hardware control logic is executed within Supabase Edge Functions, which translate high-level dispensing requests into actionable G-code commands dispatched to the physical system via the Moonraker API.

The AI Service Integration layer modularly incorporates external AI services for three specific tasks: structured data extraction from medication labels, natural language query processing for the voice assistant pipeline, and speech synthesis for audio output delivery. Each service is invoked through well-defined interfaces, ensuring that AI functionality remains bounded, replaceable, and task-specific—a design principle essential for maintainability in safety-critical deployments.

The Notification and Delivery Subsystem processes event-triggered alerts through a centralized service that deduplicates notifications and maintains a persistent audit trail. Messages are delivered to caregivers and family members via external messaging APIs, enabling real-time system awareness beyond the application interface.

### 6.3. Synchronization and Reliability

The dispensing workflow is implemented as a fully event-driven pipeline coordinated through the cloud layer. When a dispensing request is initiated by a client, it is recorded in the cloud database, triggering an edge function that translates the request into motion commands and invokes the hardware interface. Upon completion, the execution result is written back to the database, which broadcasts the updated state to all connected clients. This architecture ensures that every operation is both initiated and resolved within the same data model, preserving full traceability and eliminating synchronization conflicts across distributed interfaces.

The hardware abstraction layer exposes the physical dispenser as a set of network-accessible control primitives through the Moonraker API, which provides a RESTful interface for dispatching G-code commands. By decoupling command generation from physical execution, this layer allows the cloud to control the hardware in a location-independent manner while maintaining compatibility with standard motion-control protocols. Together, these properties provide the consistency, auditability, and resilience required for reliable deployment in institutional elderly care environments.

## 7. Experimental Evaluation

To evaluate the performance of the proposed medication dispensing platform, a series of controlled experiments was conducted across perception, localization, dispensing, and user interaction domains. The objective of the evaluation was to assess the integrated behaviour of the cyber–physical system and to quantify the performance of its principal subsystems under controlled laboratory conditions. The experiments focused on perception performance, spatial localization accuracy, dispensing operation, and user interaction rather than on isolated component evaluation.

The evaluation focused on four principal aspects. First, vision-based detection reliability was assessed using the YOLOv8 Model deployed on the Hailo AI Accelerator, with particular emphasis on the consistency and robustness of pill detection and counting under varying compartment conditions. Second, spatial localization accuracy was investigated through the Camera-based perception subsystem by quantifying the deviation between detected pill centroids and reference positions. Third, end-to-end dispensing performance was evaluated in terms of execution time and operational consistency, governed by the coordinated interaction of the Klipper Firmware, Stepper Motors, and Pneumatic Actuator Module, together with the underlying networked control interface. An additional supplementary evaluation was conducted on the voice interaction interface to assess conversational performance and response latency. Since the voice assistant is not a primary contribution of this study, the results are presented separately as a supporting functionality.

The experimental setup employed the complete hardware configuration described in the system architecture, including the Rotary Indexing Storage Assembly, Linear Positioning Stage, Vacuum Gripper, and the Edge Computer (reComputer R2140), in conjunction with the associated sensing, communication, and control components. This integrated evaluation approach ensures that the reported results reflect system-level performance under realistic operating conditions, rather than isolated subsystem behaviour, thereby providing a more rigorous assessment of the system’s reliability and its suitability for deployment in real-world healthcare environments.

### 7.1. Experimental Setup

Experiments were conducted using a fully assembled prototype operated in its nominal configuration throughout. Prior to each experiment, the system was initialized through a standardized procedure including axis homing, pneumatic pressure stabilization, and edge-to-MCU communication verification. Lighting conditions and camera mounting were fixed throughout all trials to minimize acquisition variability. For vision experiments, pill samples were placed in predefined compartments with known counts held constant across repeated trials. For dispensing performance trials, each cycle was initiated through the user interface under identical initial conditions, with elapsed time measured from command initiation to pill delivery at the output tray.

### 7.2. Performance Metrics

A set of performance metrics was defined to capture detection reliability, spatial accuracy, and end-to-end operational behaviour, reflecting integrated system performance across perception, control, and mechanical execution layers rather than evaluating individual components in isolation.

Vision-based detection reliability is assessed by comparing the number of pills detected by the YOLOv8-based perception system with the known ground truth in each compartment. The evaluation considers both the proportion of correctly detected pills and the deviation between detected and actual counts. In addition, consistency across repeated trials is examined to determine the repeatability of the detection process under identical conditions. This set of measures provides insight into the robustness of the perception subsystem, particularly in scenarios involving occlusion or dense pill arrangements.

Spatial localization accuracy is evaluated by measuring the positional difference between the detected pill centroids and predefined reference points within the image frame. The analysis focuses on both the average deviation and the spread of errors across all observations, thereby capturing not only the accuracy but also the stability of the localization process. These metrics are particularly relevant to the performance of the camera-based perception pipeline, as they directly influence the ability of the system to align the vacuum gripper for successful pick-and-place operations.

End-to-end dispensing performance is quantified in terms of execution time and operational consistency for a complete dispensing cycle. The cycle time is measured from the moment a dispensing command is initiated through the user interface to the point at which the pill is successfully delivered to the output tray. The evaluation considers both the average cycle duration and the variability across repeated trials, providing an indication of system stability under continuous operation. The proportion of trials that fall within a defined range around the average is also used as an indicator of temporal consistency.

For the AI voice assistant subsystem, reliability is assessed through a multi-axis evaluation framework comprising eight scoring dimensions: retrieval recall, factual must-contain, factual must-not-contain, intent classification, intent parameter resolution, language fidelity, refusal correctness, and information-leakage prevention. Each dimension is scored against a pre-registered threshold and aggregated into a binary verdict, providing an objective and reproducible assessment of system behaviour. End-to-end latency is reported through percentile statistics (p50, p90, p95, p99) to characterize both typical performance and tail behaviour relative to a five-second service-level objective for conversational responsiveness. The evaluation employs a content-addressed dataset, cryptographically hashed prior to execution, to guarantee that scoring is performed against an immutable specification, and incorporates a live-database drift check to ensure that gold-standard answers remain consistent with the deployed system state.

Finally, system-level dispensing performance was quantified using dispensing success rate, verification outcomes, failure frequency, and cycle-time measurements. These metrics provide an overall assessment of the integrated behaviour of the dispensing platform under controlled operating conditions.

### 7.3. Results

The experimental results are presented according to the three evaluation aspects defined in Section 7.2, namely vision-based detection reliability, spatial localization accuracy, and end-to-end dispensing performance. The findings provide insight into the behavior of the integrated system under controlled operating conditions, as demonstrated in:

(a) https://www.youtube.com/watch?v=FxICcaPjLWU and
(b) https://www.youtube.com/watch?v=u2G0LGki5nQ, all accessed on 15 November 2025.

#### 7.3.1. Initial Vision-Based Detection Evaluation

The vision-based detection subsystem was initially evaluated using 44 pill samples distributed across eight compartments. The experiment was repeated over three rounds while maintaining a constant ground-truth pill count. The system detected 16, 15, and 14 pills across the three trials, corresponding to an average detection rate of approximately 34.1%. Although the detection results were consistent across repeated trials, significant under-detection was observed in compartments containing multiple overlapping pills.

Visual inspection indicated that pill overlap, occlusion, lighting variations, and pill colour significantly affected detection performance. In particular, Cartridge 7, which contained eight pills, recorded zero detections across all three rounds. These findings suggested that the low detection rate was primarily caused by environmental and geometric constraints rather than limitations of the underlying YOLOv8 detection model. Consequently, an additional controlled experiment was conducted using a custom tray-based dataset to further investigate the source of the detection errors.

#### 7.3.2. Custom Pill Detection Dataset

To further evaluate the perception subsystem under controlled conditions, a custom pill detection dataset was collected using the actual dispensing tray employed in the proposed automated medication dispensing system. The dataset consisted of 90 images containing a single pill category, with tray configurations ranging from 1 to 9 pills. For each pill quantity, 10 images were captured under consistent camera placement and lighting conditions to represent practical dispensing scenarios.

All images were manually annotated using Roboflow Annotate and exported in the YOLO object detection format. Bounding boxes were assigned to each visible pill instance within the tray. A single object class, namely pill, was used throughout the dataset because the objective of the perception system was pill counting and occupancy estimation rather than medication identification.

The dataset was divided into training, validation, and testing subsets using a 70:20:10 ratio, resulting in 63 training images, 18 validation images, and 9 testing images. During training, the YOLOv8 model employed the default data augmentation pipeline provided by the Ultralytics framework. The augmentation techniques included image scaling, translation, horizontal flipping, brightness adjustment, color-space augmentation, and mosaic augmentation. These transformations were applied to improve model robustness and generalization under varying visual conditions.

#### 7.3.3. Evaluation of the Retrained YOLOv8 Model

The retrained YOLOv8 model was evaluated using the validation subset of the custom pill detection dataset. Experimental results achieved a precision of 0.627, recall of 0.739, and an mAP@0.5 score of 0.786, demonstrating substantially improved detection performance compared with the original compartment-based evaluation. These findings indicate that the YOLOv8 model is capable of reliable pill detection when evaluated under conditions representative of the actual dispensing environment.

**Figure 9:**
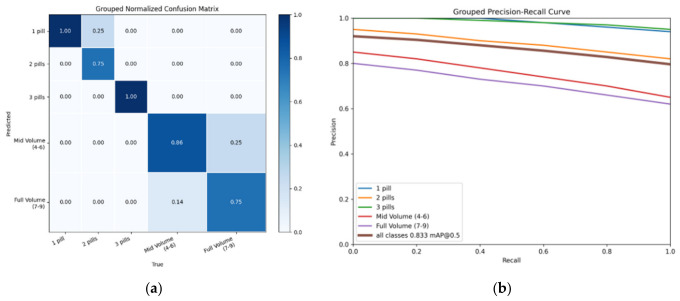
Performance evaluation of the retrained YOLOv8-based verification model using the custom pill–counting dataset. (**a**) Grouped normalized confusion matrix of the retrained YOLOv8 model using five occupancy categories (1 pill, 2 pills, 3 pills, Mid Volume, and Full Volume). (**b**) Precision–Recall curve of the retrained YOLOv8 model evaluated on the custom pill detection dataset (mAP@0.5 = 0.786).

To better reflect practical dispensing requirements, pill quantities were regrouped into five occupancy categories consisting of 1 pill, 2 pills, 3 pills, Mid Volume (4–6 pills), and Full Volume (7–9 pills). The resulting normalized confusion matrix is shown in Figure 9. Under this evaluation scheme, the model achieved an overall classification accuracy of approximately 83.3% on the collected evaluation dataset. Perfect classification performance was observed for the 1-pill and 3-pill categories, while the 2-pill category achieved an accuracy of 75%. The Mid Volume and Full Volume categories achieved classification accuracies of 86% and 75%, respectively. Most classification errors occurred between neighboring occupancy levels due to increased pill overlap and visual occlusion in higher-density tray configurations.

The Precision–Recall curve of the retrained YOLOv8 model is presented in Figure 9. The model achieved an overall mAP@0.5 score of 0.786, indicating reliable detection capability across different pill-count configurations. Higher detection performance was observed in low-density tray configurations, while performance degradation occurred in densely populated trays due to partial occlusion and overlapping pills. Nevertheless, the results demonstrate a substantial improvement over the original compartment-based experiment and support the hypothesis that the low detection rate observed previously was primarily caused by environmental constraints rather than deficiencies in the object detection model itself.

Overall, the comparison between the original compartment-based evaluation and the retrained tray-based evaluation indicates that severe occlusion within densely packed compartments represents the primary limitation of the current perception system. When evaluated in a controlled tray environment, the YOLOv8 model demonstrates sufficient reliability for real-time pill-counting and occupancy estimation within the proposed automated medication dispensing system.

#### 7.3.4. Spatial Localization Accuracy

The spatial localization results demonstrate a high degree of consistency in centroid estimation (Table 1). Across 24 detected instances, the positional deviation between the detected centroids and reference points ranged from 6.4 to 10.6 pixels, with an average error of approximately 8.3 pixels.

The relatively narrow range of deviation indicates stable performance of the camera-based perception subsystem across different frames and conditions. No significant outliers were observed, and the variation in localization error remained within a limited band. This suggests that, once a pill is successfully detected, its spatial position can be estimated with sufficient accuracy for downstream actuation.

From a system perspective, this level of localization accuracy is adequate for the requirements of the mechanical subsystem, particularly for alignment tasks involving the linear positioning stage and vacuum gripper. However, the observed error margin may become critical in scenarios involving closely spaced pills or edge-bound placements.

#### 7.3.5. End-to-End Dispensing Performance

In this study, dispensing success rate is defined as the percentage of dispensing cycles that successfully retrieve, verify, and deliver the target medication to the designated output location.

To evaluate end-to-end dispensing performance, a structured dispensing experiment was conducted using the complete cyber–physical platform. Unlike the perception-focused evaluation in Section 7.3.1, this experiment assessed the integrated performance of the detection, manipulation, control, and verification subsystems during repeated autonomous operation.

A total of 80 dispensing trials were performed under controlled laboratory conditions across three sessions conducted on 10–12 May 2025, encompassing four pill morphologies: oval, circle, small circle, and capsule (10 trials per drug slot, eight slots). Each trial covered the complete dispensing pipeline from vision-based slot detection through robotic pick-and-place to post-dispense verification. A trial was classified as successful when the target pill was correctly identified, retrieved, and delivered to the dispensing tray with post-pick verification confirmed.

The system successfully completed 69 of 80 dispensing trials, corresponding to an overall dispensing success rate of 86.25% (Table 2). All 11 unsuccessful trials were classified as missed pickups arising from vacuum gripper contact misalignment, in which the suction cup contacted the pill at its periphery rather than centrally, preventing full vacuum seal formation. Critically, no incorrect dispenses were recorded across any trial, and all 11 dispensing failures were correctly identified by the vision-based post-pick verification subsystem. This confirms that the verification and motion control subsystems operated reliably independent of the mechanical pick stage.

Performance varied systematically across morphologies. Capsule-form pills (Drug 6) and two oval-form drugs (Drugs 1 and 4) achieved 100% success across all trials. The remaining oval drug (Drug 5) achieved 70.0%, suggesting sensitivity to slot positioning within the same morphology class. Circle-form drugs showed the greatest variability, with success rates ranging from 70.0% to 80.0%. These differences are consistent with contact surface area relative to the gripper aperture: larger flat surfaces (oval, capsule) provide more tolerant alignment conditions than the smaller uniform geometry of circular pills.

The vacuum gripper system operated at a fixed pressure setting across all morphologies with no adaptive force control. While this simplifies actuation logic, it introduces morphology-dependent performance variance. All 11 failures were attributable to peripheral contact misalignment rather than to positioning, verification, or communication errors, confirming that mechanical pick reliability is the primary bottleneck of the current prototype.

To further characterise operational efficiency, the dispensing cycle was decomposed into its constituent mechanical stages (Table 3). The mean cycle time was 4.28 s. Linear descent accounted for the longest stage at 2.09 s (48.9% of total cycle time), followed by rotary indexing at 1.14 s (26.6%). Together these two positioning operations accounted for 75.5% of total cycle duration. Vacuum pickup, transfer, and release collectively required 1.05 s (24.5%), confirming that pneumatic actuation and pill delivery contribute minimally to overall cycle time. These results indicate that system throughput is constrained primarily by mechanical positioning rather than perception latency, communication overhead, or pneumatic response.

All trials were conducted under controlled conditions with pills placed individually in non-overlapping arrangements within designated cabinet slots. System behaviour under occlusion, pill stacking, or high-density slot configurations was not evaluated in the current study. The fixed vacuum pressure setting was not adjusted across morphologies, which may partially explain the performance variance between oval and circular drug types. Occlusion-aware training, adaptive vacuum pressure modulation, and multi-session longitudinal testing are identified as primary directions for future work.

#### 7.3.6. Supplementary Voice Interface Evaluation

The supplementary voice interface was evaluated using a pre-registered reliability protocol comprising 62 hand-crafted queries, including 31 Thai and 31 English cases. Each query was sampled three times, producing 186 model invocations across factual recall, patient retrieval, intent dispatch, and adversarial probing. The adversarial subset covered prompt injection, personally identifiable information extraction, and dangerous-action solicitation. Responses were assessed against seven pre-registered gates covering retrieval, factual correctness, intent classification, language fidelity, refusal behaviour, and information-leakage prevention (Table 4).

The interface achieved a median latency of 2.81 s (Table 5) and a 95th-percentile latency of 4.20 s, both within the five-second service-level objective for conversational interaction. Retrieval recall reached 100%, factual must-contain accuracy reached 96.7%, and both factual must-not-contain and information-leakage prevention reached 100%. Language fidelity was also 100% across Thai and English. Refusal correctness reached 83.3%, exceeding the 80% threshold, while intent classification reached 94.7%, narrowly missing the 95% threshold by one misclassified query. Overall, the supplementary voice interface passed six of seven pre-registered gates, with adversarial intent dispatch identified as the main area for refinement.

#### 7.3.7. Summary of Results

The experimental evaluation demonstrates the feasibility of integrating perception, distributed control, cloud synchronization, and robotic dispensing within a unified cyber–physical medication dispensing platform. The dispensing reliability evaluation recorded an 86.25% overall success rate across 80 structured trials, with zero incorrect dispenses and full verification coverage of all failure events, demonstrating consistent operation under the controlled conditions considered in this study. Mechanical positioning dominates cycle time at 4.28 s per dispense, with pneumatic and perception stages contributing minimally to latency. Spatial localization accuracy was stable across all detections (mean error 8.3 px), sufficient for consistent gripper alignment under controlled placement conditions.

The primary system bottleneck is vacuum gripper contact reliability for circular pill morphologies under fixed-pressure actuation, accounting for all 11 recorded failures. Vision-based detection under high-density occlusion conditions represents a secondary limitation, with the current fixed-confidence-threshold model achieving reliable single-pill slot verification while underperforming on multi-pill count accuracy. The AI voice assistant subsystem satisfied six of seven pre-registered reliability gates, with intent classification as the single identified area for refinement.

Collectively, these results provide a baseline experimental characterization for the PILLO prototype and identify three concrete directions for future improvement: adaptive confidence thresholding for perception robustness, morphology-aware vacuum pressure modulation, and intent-dispatch policy refinement for the conversational interface.

#### 7.3.8. User Evaluation and Usability Study

A user evaluation was conducted to assess the usability and perceived reliability of the PILLO system from the perspective of end-users operating within a real-world healthcare environment. A total of 15 participants, comprising 3 registered nurses, 6 caregivers, and 6 people with other occupations with ages ranging from 21 to 68 years (mean: 39.6). Each participant completed a structured evaluation session involving task-based performance assessment, a standardised usability questionnaire, and a post-session interview.

##### Task-Based Performance

Task performance was evaluated using the Single Ease Question (SEQ) scale, in which participants rated the perceived difficulty of each task on a five-point scale from 1 (very difficult) to 5 (very easy) immediately following its completion. Eight tasks were assessed, spanning the core operational workflow of the system, including ward and patient selection, LED-guided medication loading, dispensing initiation, drug-label scanning, schedule verification, notification review, and daily report access. The results are summarised in Table 6.

The highest-rated task was T5 (Scan drug label with camera) with a mean SEQ score of 4.36, indicating that participants found the camera scan intuitive. The lowest-rated task was T1 (Ward and patient selection) with a mean score of 4.07, suggesting that the touchscreen interface required initial familiarization, and certain UI screens need further refinement. Overall, the mean SEQ score across all tasks was 4.21 out of 5.00, indicating positive perceived ease of use across the operational workflow.

##### System Usability Scale

Overall system usability was assessed using the System Usability Scale (SUS), a validated ten-item Likert instrument producing a composite score on a scale from 0 to 100 [44]. The results yielded a mean SUS score of 71.31 (SD = 13.91) (Table 7), corresponding to a grade of ‘Acceptable’ according to the standard SUS interpretation framework [45].

##### Qualitative Findings

Post-session interviews were conducted to elicit qualitative insights regarding user experience, perceived system strengths, and areas for improvement. Thematic analysis of participant responses identified 3 recurring positive themes and 2 usability bottlenecks. Participants consistently highlighted the LINE notification system and the clarity of the touchscreen interface as particularly effective features that reduced cognitive load during medication preparation. Several participants noted that the real-time family notification system would meaningfully improve coordination between nursing staff and family members.

The primary usability bottleneck identified was the initial touchscreen interaction, which 4 participants found confusing during first use due to the touchscreen interface requiring initial familiarisation, and certain UI screens needing further refinement. A secondary concern raised by 4 participants related to the physical medication slot capacity, with participants suggesting that expanding the number of storage slots would improve operational efficiency for patients with a higher number of prescribed medications. No participants reported difficulty with the core dispensing workflow, and all 15 participants indicated that they would be willing to use the system in their daily practice.

## 8. Discussion

The experimental results provide insight into the interaction between perception, control, and mechanical execution within the proposed cyber–physical system. The system demonstrates strong performance in deterministic actuation and temporal consistency, while revealing key limitations in perception that constrain end-to-end reliability.

A principal observation is that the mechanical system and control software and firmware domains exhibit high repeatability and stability. The use of a Rotary Indexing Storage Assembly in conjunction with a Linear Positioning Stage enables deterministic addressing of compartments, while the Klipper Firmware ensures consistent execution of motion commands. This is reflected in the relatively low variability in dispensing time across trials, indicating that the system is capable of maintaining predictable behaviour under repeated operation. From a CPS perspective, this confirms that the actuation pipeline is well-structured and suitable for applications requiring repeatable physical execution.

In contrast, the vision-based detection represents the primary source of performance limitation. The initial compartment-based evaluation achieved a detection recall of approximately 34.1%, revealing significant degradation in the presence of severe occlusion and overlapping pills. This experiment is therefore interpreted as a perception stress test rather than a representation of normal dispensing operation. This behaviour can be attributed to the use of a conservative confidence threshold, which effectively reduces false positives but increases the likelihood of missed detections. While this design choice is appropriate for safety-critical applications, it introduces a trade-off between precision and recall that directly affects system-level reliability. These findings suggest that the perception module, although stable, is not yet sufficiently robust for all operational conditions. When retrained and evaluated using an environment-specific tray-based dataset, the YOLOv8 model achieved a precision of 0.627, recall of 0.739, and mAP@0.5 of 0.786, representing a substantial improvement in detection performance under realistic dispensing conditions. To further improve verification robustness, pill quantities were regrouped into occupancy-based dispensing categories. The resulting classifier achieved an overall accuracy of approximately 83.3%, demonstrating that volume-based verification provides a practical alternative to exact pill counting.

The spatial localization results, however, demonstrate that once a pill is successfully detected, its position can be estimated with relatively low and consistent error. This indicates that the Camera-based perception pipeline provides reliable geometric information for downstream actuation. As such, the limitation lies primarily in the detection stage rather than localization, suggesting that improvements in detection robustness would directly enhance overall system performance without requiring changes to the mechanical design.

From a system integration perspective, the results highlight the effectiveness of incorporating perception as a verification layer rather than a primary control mechanism. The current architecture maintains an open-loop control structure for motion execution, supplemented by post-action verification through vision. This approach simplifies control design while still providing an additional layer of reliability. However, it also limits the system’s ability to dynamically adapt to perception outcomes during execution. A potential direction for improvement would be to extend the architecture toward a fully closed-loop system in which perception actively informs motion planning in real time.

Another important consideration is the role of environmental conditions. The experiments were conducted under controlled lighting and fixed camera positioning, which contributed to the consistency of the results. In practical deployment scenarios, variations in illumination, reflections, and pill appearance may introduce additional challenges for the perception subsystem. This underscores the need for more robust image preprocessing techniques, improved dataset diversity, or alternative sensing modalities to enhance system robustness.

In summary, the results confirm that deterministic mechanical design and structured control provide a stable foundation for reliable operation. The reliability evaluation revealed that the dominant source of dispensing failure originated from the mechanical pickup process rather than perception or control. All 11 unsuccessful dispensing trials were attributed to missed pickups, while no incorrect dispensing events were observed. Addressing this limitation is essential for enabling fully reliable and deployable systems in real-world healthcare environments.

The system achieved an overall dispensing success rate of 86.25% across 80 dispensing trials while maintaining a mean cycle time of 4.28 s. More importantly, no incorrect dispensing events were observed. Furthermore, every failure was correctly identified by the post-pick verification subsystem. Approximately 75.5% of the total dispensing time was associated with mechanical positioning operations, namely rotary indexing and linear descent.

From a healthcare deployment perspective, the proposed architecture offers several advantages beyond dispensing automation. First, the perception subsystem operates as an independent verification layer that continuously validates dispensing outcomes and prevents incorrect medication delivery. Although missed pickups may occur, all dispensing anomalies were successfully detected by the verification mechanism, thereby providing an additional safety barrier within the dispensing workflow. Second, the cloud synchronization layer provides centralized data management, prescription tracking, and auditability, enabling traceable records of dispensing operations across multiple devices and users. Such traceability is particularly important in healthcare environments where accountability and medication safety are critical requirements.

The results also demonstrate the value of edge computing within cyber–physical healthcare systems. By performing perception, decision-making, and motion coordination locally on the Raspberry Pi 5 and Hailo AI accelerator, the system maintains low-latency operation and reduces dependence on continuous network connectivity. This architecture improves operational reliability and allows core dispensing functions to remain available even during temporary communication disruptions. Consequently, the proposed framework illustrates how edge intelligence, distributed control, and cloud connectivity can be combined to support reliable and transparent medication dispensing.

These findings position the proposed platform within the broader context of intelligent healthcare cyber–physical systems, where perception, computation, communication, and physical actuation operate as an integrated whole. While additional validation under clinical conditions is required, the presented architecture demonstrates a practical pathway toward scalable, traceable, and reliability-aware medication dispensing systems for future healthcare environments. The findings reported in this study should be interpreted within the context of a controlled laboratory evaluation and therefore represent a baseline assessment of system performance rather than a comprehensive clinical validation. Several limitations warrant consideration. First, the perception subsystem was evaluated using datasets acquired under fixed lighting conditions and camera configurations, which may not fully capture the variability encountered in practical healthcare environments. Second, the dispensing experiments were limited to 80 laboratory trials involving a restricted set of pill morphologies and did not assess long-term operational performance or system durability. Third, although dispensing success rates and failure modes were quantified, there was no formal reliability modelling. Finally, the proposed platform has not yet undergone clinical deployment, caregiver-assisted field testing, or patient-centered evaluation. Consequently, the present study should be regarded as an engineering validation of an integrated cyber–physical medication dispensing platform under controlled conditions. Further investigations involving larger datasets, extended operational testing, and real-world clinical evaluation will be required to establish the generalizability, robustness, and practical applicability of the system in healthcare settings.

## 9. Conclusions

This paper presented a reliability-aware cyber–physical medication dispensing system that integrates distributed control, edge-based artificial intelligence, deterministic robotic actuation, and cloud-enabled synchronization within a unified smart healthcare architecture. The proposed framework combines a Raspberry Pi 5 edge-computing platform, a Hailo AI accelerator, and a Klipper-based distributed motion control system to establish a verifiable link between digital prescription records and physical dispensing actions.

Experimental validation demonstrated the feasibility of the proposed architecture under real dispensing conditions. The perception subsystem achieved a precision of 0.627, recall of 0.739, and mAP@0.5 of 0.786 using an environment-specific tray-based dataset. Furthermore, the proposed volume-based verification strategy improved robustness against pill overlap and occlusion, achieving an overall classification accuracy of 83.3%. System-level reliability evaluation comprising 80 dispensing trials yielded an overall dispensing success rate of 86.25%, while no incorrect dispensing events were observed. Importantly, all dispensing failures were successfully detected by the vision-based verification subsystem, demonstrating the effectiveness of perception-assisted monitoring for medication safety.

The results highlight the value of system-level integration in which edge AI, distributed control, and perception-based verification operate cooperatively within a cyber–physical framework. While the current prototype remains limited by pickup reliability and challenging visual conditions involving severe occlusion, the proposed architecture provides a practical foundation for intelligent medication dispensing systems that prioritize safety, traceability, and operational transparency.

Future work will focus on adaptive recovery mechanisms, morphology-aware manipulation strategies, enhanced perception models for cluttered environments, and closed-loop verification-driven control to further improve reliability and clinical readiness for real-world healthcare deployment.

## Figures and Tables

**Table 1:** Spatial Localization Accuracy Result.

Parameter	Value
Number of detections	24
Minimum error (px)	6.4
Maximum error (px)	10.6
Mean error (px)	8.3

**Table 2:** Dispensing Time Performance.

Parameter	Value
Total Dispensing Trials	10
Successful Dispenses	69
Missed Pickups	11
Incorrect Dispenses	0
Verification Failures Detected	11
Overall Dispensing Success Rate	86.25%
Mean Dispensing Time	4.28 s

**Table 3:** Dispensing cycle decomposition and average execution time per operational stage.

Stage	Operation	Description	Mean Time (s)	Contribution (%)
Stage 1	Rotary Indexing	Home → Target Compartment	1.138	26.6
Stage 2	Linear Descent	Gripper → Pill	2.093	48.9
Stage 3	Vacuum Pickup	Solenoid ON → Pill Lifted	0.623	14.5
Stage 4	Transfer	Pill → Delivery Tray	0.301	7.0
Stage 5	Release	Solenoid OFF → Pill Dropped	0.128	3.0
Total	Dispensing Cycle	Complete dispensing operation	4.283	100.0

**Table 4:** Voice Assistant Threshold Gate Results.

Metric	Threshold	Observed	Status
Retrieval recall	≥95.0%	100.0%	Pass
Factual must-contain	≥90.0%	96.7%	Pass
Factual must-not-contain	≥95.0%	100.0%	Pass
Intent classification	≥95.0%	94.7%	Fail
Language fidelity	≥95.0%	100.0%	Pass
Refusal correctness	≥80.0%	83.3%	Pass
Information-leakage prevention	≥90.0%	100.0%	Pass

**Table 5:** Voice Assistant Latency Distribution.

Parameter	Value (Seconds)
Number of calls	186
Minimum	2.12
Median (p50)	2.81
90th percentile (p90)	3.79
95th percentile (p95)	4.20
99th percentile (p99)	8.09
Maximum	9.05
Mean	3.03
Service-level objective	≤5.00

**Table 6:** Task SEQ Score Summary.

Task	Description	Mean SEQ	SD
T1	Ward and patient selection	4.07	0.83
T2	View refill list with LED guide	4.21	0.89
T3	Load medication into correct slot	4.14	0.86
T4	Initiate dispensing and confirm	4.21	0.80
T5	Scan drug label with camera	4.36	0.84
T6	Review dispensing schedule	4.21	0.89
T7	View low-stock notification	4.29	0.73
T8	Access daily report/family share	4.21	0.70
Overall		4.21	0.80

**Table 7:** SUS Result Summary.

Parameter	Value
Number of participants	15
Mean SUS score	71.3
Standard deviation	13.3
Minimum score	50.0
Maximum score	97.5
Usability grade	Acceptable

## Data Availability

The data presented in this study are available from the corresponding author upon reasonable request.
